# Association of p53 codon72 Arg>Pro polymorphism with susceptibility to nasopharyngeal carcinoma: evidence from a case–control study and meta-analysis

**DOI:** 10.1038/oncsis.2016.31

**Published:** 2016-05-09

**Authors:** S K Sahu, S Chakrabarti, S D Roy, N Baishya, R R Reddy, S Suklabaidya, A Kumar, S Mohanty, S Maji, A Suryanwanshi, S Rajasubramaniam, M Asthana, A K Panda, S P Singh, S Ganguly, O P Shaw, A K Bichhwalia, P K Sahoo, N R Chattopadhyay, K Chatterjee, C N Kundu, A K Das, R Kannan, Zorenpuii, E Zomawia, S A Sema, Y I Singh, S K Ghosh, K Sharma, B S Das, T Choudhuri

**Affiliations:** 1 Division of Infectious Disease Biology, Institute of Life Sciences, Bhubaneswar, India; 2 Department of Radiation Oncology, Civil Hospital, Dimapur, Nagaland, India; 3 Dr B. Borooah Cancer Institute, ENT Department, Guwahati, Assam, India; 4 Department of Biotechnology, Regional Medical Research Centre for Tribals, Jabalpur, India; 5 Central University of Jharkhand, Brambe, Ranchi, India; 6 Shri Ramachandra Bhanj Medical College, Cuttack, Department of Gastroentrology, Odisha, India; 7 Department of Biotechnology, Siksha Vhabana, Visva Bharati, Santiniketan, Bolpur, India; 8 School of Biotechnology, KIIT University, Bhubaneswar, India; 9 Cachar Cancer Hospital & Research Centre, Silchar, Assam, India; 10 Civil Hospital, Aizawl, Mizoram, India; 11 Regional Institute of Medical Sciences, Department of Radiotherapy, Imphal, Manipur, India; 12 Department of Biotechnology, Assam Central University, Silchar, India; 13 Guwahati Medical College & Hospital, Guwahati, Assam, India

## Abstract

Tumor suppressor p53 is a critical player in the fight against cancer as it controls the cell cycle check point, apoptotic pathways and genomic stability. It is known to be the most frequently mutated gene in a wide variety of human cancers. Single-nucleotide polymorphism of p53 at codon72 leading to substitution of proline (Pro) in place of arginine (Arg) has been identified as a risk factor for development of many cancers, including nasopharyngeal carcinoma (NPC). However, the association of this polymorphism with NPC across the published literature has shown conflicting results. We aimed to conduct a case–control study for a possible relation of p53 codon72 Arg>Pro polymorphism with NPC risk in underdeveloped states of India, combine the result with previously available records from different databases and perform a meta-analysis to draw a more definitive conclusion. A total of 70 NPC patients and 70 healthy controls were enrolled from different hospitals of north-eastern India. The p53 codon72 Arg>Pro polymorphism was typed by polymerase chain reaction, which showed an association with NPC risk. In the meta-analysis consisting of 1842 cases and 2330 controls, it was found that individuals carrying the Pro allele and the ProPro genotype were at a significantly higher risk for NPC as compared with those with the Arg allele and the ArgArg genotype, respectively. Individuals with a ProPro genotype and a combined Pro genotype (ProPro+ArgPro) also showed a significantly higher risk for NPC over a wild homozygote ArgArg genotype. Additionally, the strength of each study was tested by power analysis and genotype distribution by Hardy–Weinberg equilibrium. The outcome of the study indicated that both allele frequency and genotype distribution of p53 codon72 Arg>Pro polymorphism were significantly associated with NPC risk. Stratified analyses based on ethnicity and source of samples supported the above result.

## Introduction

Nasopharyngeal carcinoma (NPC) arises from the epithelial cells that cover the upper part of the throat behind the nose and near the base of the skull. The disease is treatable at an early stage but the majority of NPC patients are diagnosed at a late stage because of the exhibition of nonspecific symptoms related to other head and neck illnesses.^1, 2^ General symptoms of NPC include trismus, otitis media, hearing loss, nasal regurgitation, cranial nerve palsies, nasal twang, bleeding and pain.^3^ The World Health Organization histopathological grading system classifies NPC into three types: keratinizing squamous cell carcinoma; non-keratinizing differentiated carcinoma; and undifferentiated carcinoma.^4^ The American joint committee on cancer established tumor, node and metastasis classification to determine the different stages of NPC.

Epidemiological studies suggest the association of food habits (alcohol, intake of salted fish containing nitrosamine, herbal tea and herbal medicine), lifestyle (occupational exposure to formaldehyde, chlorophenol, wood dust, tobacco users) and viral infection (Epstein–Barr virus and human papilloma virus) in the etiology of NPC.^5, 6, 7, 8, 9^ However, many individuals exposed to these parameters do not develop NPC, which indicates the involvement of genetic factors. To establish a link between genetic factors and NPC development, study of single-nucleotide polymorphism (SNP) in tumor suppressor genes has been the focus of many researchers.

p53 is a well-established tumor suppressor gene located on chromosome 17p13.1. It plays a critical role in response to genotoxic stress and tries to maintain genomic stability and control proper execution of the cell cycle and apoptotic pathways.^10, 11, 12^ Deregulated function of p53 may result in loss of this regulation, resulting in uncontrolled cell proliferation and cancer development.^13, 14, 15^ Polymorphisms in p53 or target genes impair the function of the p53 signaling pathway.^16^ The most studied polymorphism in p53 is located in exon 4 at codon72. It carries either the CGC sequence that encodes arginine or the CCC sequence that encodes proline due to G/C transversion.^17, 18^ As a result, two allelic forms (Arg and Pro) and three genotypes (ArgArg, ArgPro and ProPro) have evolved. These allelic variants and genotypes oscillate in their binding capacity to the transcriptional factors, induction of apoptosis and repression of transformation of human cells.^7, 18, 19, 20^ Arg variants induce apoptosis more efficiently than do the Pro variants, which may be due to their ability to localize into mitochondria and regulate the release of cytochrome *C* into cytosol.^18^ The released cytochrome *C* in turn activates caspase-3, one of the key executioners of apoptosis.^21, 22^ This difference between Arg and Pro variants may provide the plausible cause for Pro allele's involvement in increased susceptibility to NPC. Earlier, several studies including our present study among the populations of north-eastern India have investigated the relation between p53 codon72 Arg>Pro polymorphism and NPC risk.^23, 24, 25, 26, 27, 28, 29, 30, 31, 32^ The purpose of a case–control study of north-eastern Indian populations was also to find out the incidence of different stages of NPC among them and to examine the clinical symptoms manifested by them. However, these findings were inconsistent and inconclusive. In view of the fact that a single study may have been underpowered in clarifying the association, we performed a meta-analysis to combine the findings of all earlier studies from public records and data from the present study according to PRISMA (Preferred Reporting Items for Systematic reviews and Meta-Analyses)^33^ guidelines to explore the overall association and derive a near-specific conclusion.

## Results and discussion

NPC is a public health problem in many countries; it has a complex etiology and ranks 24th among the most frequently diagnosed cancers.^34^ The incidence rate of this cancer is highest in south-east Asia and about 92% of new cases are being found in economically developing countries.^34^ In India, this rate is comparable to that of the United Kingdom with the younger age peak in the second decade.^35, 36^ Several susceptible genes have been implicated for NPC risk, such as tumor suppressor p53, TGFβ1, IL-12 p40 and DNA repair genes.^28, 37, 38, 39^ In contrast, FokI and Bsm I polymorphisms of vitamin D receptor gene, SNP of deleted in liver cancer-1 (−29A/T) showed no association with NPC.^40, 41^ However, polymorphisms in PIN-1, TNF-α and glutathione *S*-transferase genes are indirectly associated with NPC as they influence the p53 codon72 polymorphism.^42, 43, 44^ These studies suggest that genetic predisposition may play a role in NPC development. Hence, we conducted a study in the north-eastern Indian population among healthy controls and NPC patients to find out the prevalence of p53 codon72 Arg>Pro polymorphism (Table 1). In the control population, the wild-type homozygous ArgArg genotype (48.57%) was more prevalent than the mutant heterozygous ArgPro genotype (28.57%) and mutant homozygous ProPro genotype (22.86%). Also, the prevalence of the Arg allele (62.86%) was higher than that of the Pro allele (37.14%). In the NPC population, the ArgPro genotype (47.14%) was encountered more than the ArgArg genotype (20%) and the ProPro genotype (32.86%). However, the Pro allele (56.43%) was more prevalent than the Arg allele (43.57%). The allele and genotype frequencies of the p53 codon72 polymorphism observed in this population were comparable to those of previous studies conducted in other populations.^23, 24, 25, 26, 27, 28, 29, 30, 31, 32^ Significant association was observed in the distribution of p53 codon72 polymorphism in controls and NPC patients (Pro allele: *P*=0.001, OR=0.45, 95% CI=0.28–0.73; ArgPro genotype: *P*=0.03, OR=0.39, 95% CI=0.18–0.97; ProPro genotype: *P*=0.008, OR=0.28, 95% CI=0.11–0.69) (Table 2).

The p53 codon72 SNP has been studied by many groups across the world. In the Chinese population Birgander *et al.*, in 1996, and Yung WC *et al.*, in 1997 demonstrated no association between the mutant p53 codon72 and the risk for NPC.^23^ Subsequently, Tsai *et al.*,^24^ in 2002, reported that the p53 ProPro homozygote was a risk factor for NPC development. In the Thai population, Tiwawech *et al.*,^25^ in 2003, reported that the p53 gene polymorphism may be associated with NPC susceptibility, particularly the Pro/Pro genotype carriers in subjects older than 40 years. In Portugal, Sousa *et al.*^26 ^ in 2006 reported similar findings linking the susceptibility of the P53 codon72 polymorphism to NPC. Further, Hadhri-Guiga *et al.*,^27^ in 2007, found that individuals carrying the ProPro homozygote carried an elevated risk for NPC in Tunisia. Similarly, Xiao M *et al.*,^29^ in 2010, found that the p53 codon72 polymorphism carried an increased NPC risk independently or in combination with the murine double minute-2 (MDM2) polymorphism in a Chinese population sample, suggesting a gene–gene interaction in NPC pathogenesis. Furthermore, Li *et al.*,^28^ in 2013, reported that p53 codon72 and miR-34b/c rs4938723 polymorphisms may singly or collaboratively contribute to the risk for NPC. Two more studies reported this polymorphism as an independent prognostic marker for NPC, and hence one could speculate that this polymorphism means more risk for incidence and more risk for an aggressive disease.^45, 46^ Moreover, Zhang *et al.*, in 2014, observed a weak effect of p53 polymorphisms on NPC risk. However, they found a significant risk with combination genotypes (i.e., p53 codon72 ArgPro+ProPro, MDM2 rs2279244 GT+GG, PTEN rs11202592 CC, AKT1 rs1130233 AA).^30^ Overall, variability in study results may be attributed to variation in study design, environmental factors, genetic backgrounds, racial heterogeneity, sample size, source of controls and enrollment criteria for NPC cases. A previous meta-analysis showed that the ProPro homozygote of p53 codon72 possesses an increased NPC risk.^47^ In another meta-analysis, Jiqiao Yang *et al.*^48^ analyzed publicly available data under five comparison models (allele contrast, homozygous, heterozygous, dominant and recessive) and showed the association of p53 codon72 Arg>Pro, MMP-1 (1G>2G), MMP-2 (−1306C>T), CYP2E1 (RsaI) and XRCC1 codon399 Arg>Gln polymorphism with increased risks for NPC.

**Figure 1:**
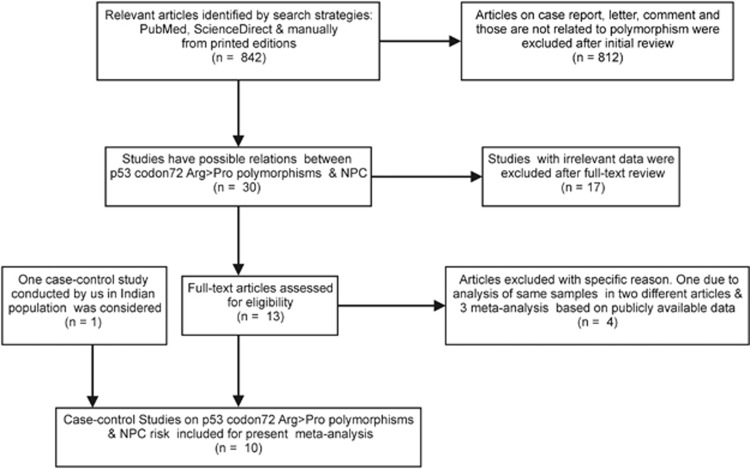
Flow chart showing the overall process of the study identification and selection. All case–control studies related to p53 codon72 and NPC were searched for in Pub Med, Science direct and manually from printed editions in different journals published up to December 2015. The search items included the combination of the following key words: p53, p53 codon72, p53 codon72 ArgPro, p53 codon72 Arg>Pro, p53 Arg72Pro or rs1042522; and nasopharyngeal cancer, nasopharyngeal carcinoma or NPC; and mutation, polymorphism, single nucleotide polymorphisms or SNPs. The inclusion criteria were case–control studies in peer-reviewed journals and articles containing useful allele and genotype frequency. The exclusion criteria were case reports without control, overlapping data with previous publications, and review articles.

In our meta-analysis, all eligible reports that fulfilled the inclusion criteria were identified from publication search and the data from the north-eastern Indian population were also included for evaluation.^23, 24, 25, 26, 27, 28, 29, 30, 31, 32^ Thus, a total of 10 case–control studies counting 1842 NPC patients and 2330 controls comprising populations from India, China, Tunisia, Portugal and Thailand were included in the final meta-analysis (Figure 1). The characteristics of all studies considered for the p53 codon72 Arg>Pro polymorphism were given. Minor allele frequency, Hardy–Weinberg equilibrium and a *post hoc* power of each study were calculated to detect the probability of association between p53 codon72 Arg>Pro polymorphisms and NPC at the 0.05 level of significance, assuming small effect size (*w*=0.15). In the north-eastern Indian population, the minor allele frequency of the p53 codon72 Arg>Pro was 0.37 for controls and 0.56 for NPC. The power of this case–control study was too weak (23%) to detect any mild effect of the polymorphisms on disease susceptibility.

The distribution of genotype frequency among controls in all these studies did not deviate from Hardy–Weinberg equilibrium since *P*>0.05, except the study in the north-eastern Indian population (Table 3).

It is worth noting that the small size of samples from the north-eastern population may be due to the low incidence of NPC.

**Figure 2:**
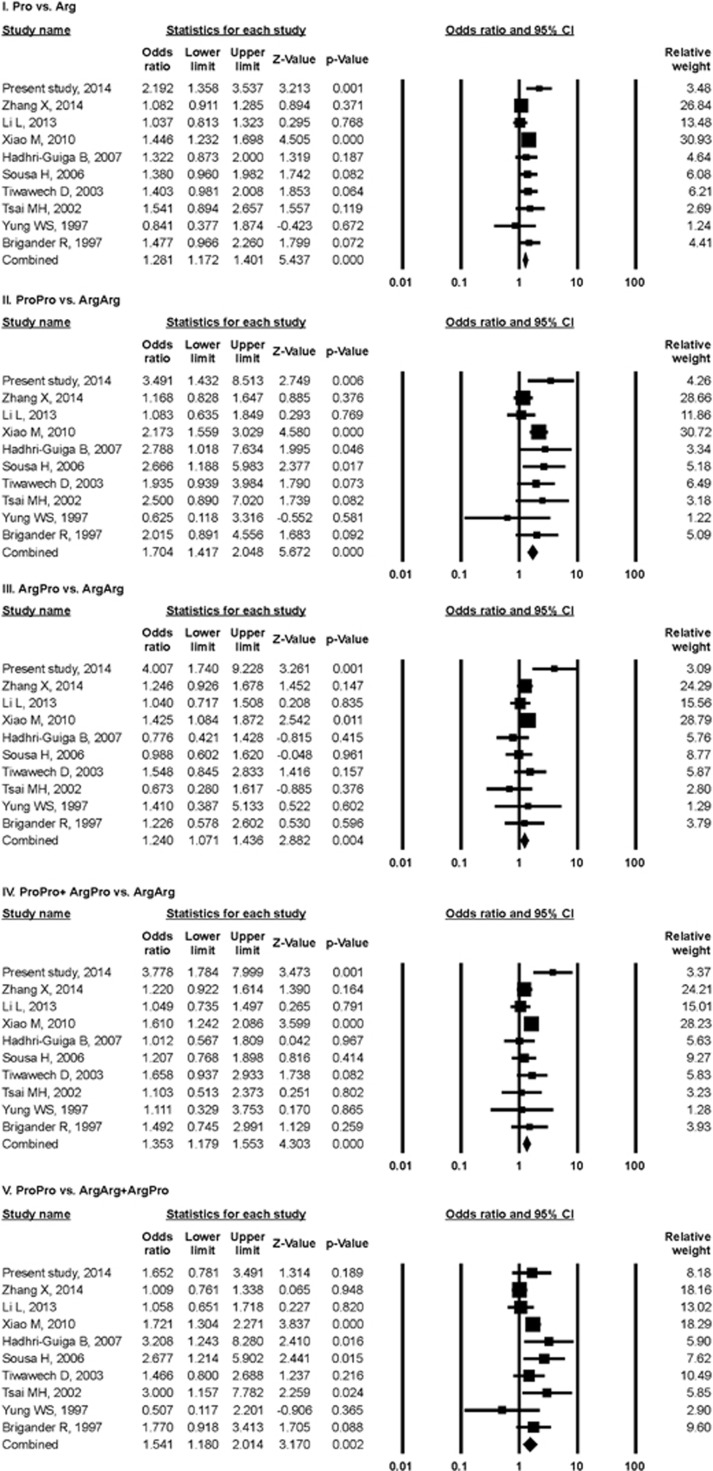
Forest plots for association between p53 codon72 Arg>Pro polymorphism and NPC risk. The squares and horizontal lines correspond to the study-specific OR and 95% CI, respectively. The area of the squares reflects the study-specific weight and the diamond represents the pooled OR and 95% CI.

**Figure 3:**
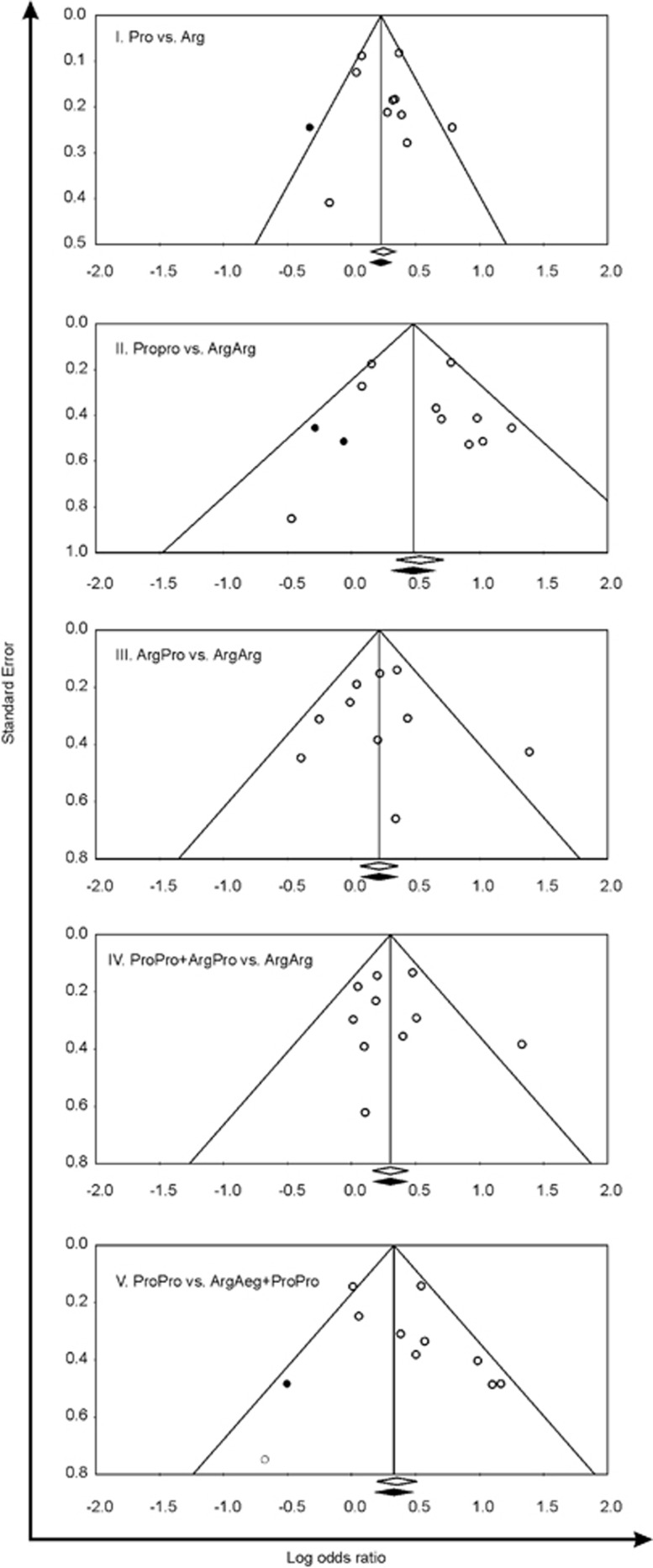
Funnel plots of Egger's test to detect publication bias. Each point represents a separate study. The OR was plotted on a logarithmic scale against the precision of each study.

Significant associations between p53 codon72 Arg>Pro polymorphism and NPC risk were observed in the combined analysis of overall studies (Pro vs Arg: OR=1.28, 95% CI=1.17–1.40, *P*_OR_ <0.001; ProPro vs ArgArg: OR=1.70, 95% CI=1.41–2.04, *P*_OR_<0.001; ArgPro vs ArgArg: OR=1.24, 95% CI=1.07–1.43, *P*_OR_=0.004; ProPro+ArgPro vs ArgArg: OR=1.35, 95% CI=1.17–1.55, *P*_OR_<0.001; ProPro vs ArgArg+ProPro: OR=1.54, 95% CI=1.18–2.01, *P*_OR_=0.002) (Figure 2). Stratified analysis was performed according to ethnicity (Asian, Caucasian), source of sample (hospital-based and population-based studies) and in Chinese studies. The pooled ORs (Table 4) and forest plots (figures not shown) indicated that the p53 codon72 polymorphism among Asians and population-based studies was associated with the development of NPC in all five comparison models (Pro vs Arg, ProPro vs ArgArg, ArgPro vs ArgArg, ProPro+ArgPro vs ArgArg and ProPro vs ArgArg+ProPro). In Caucasian and hospital-based studies a similar risk was noted in three comparison models (Pro vs Arg, ProPro vs ArgArg and ProPro vs ArgArg+ProPro). In the overall Chinese studies NPC risk was found for all comparison models except for the recessive model. Sensitivity analyses were carried out to assess the stability of the results in the overall and stratified analysis by sequential omission of individual study each time. It was observed that the influence of individual data sets on the significance of pooled ORs was not markedly influenced by any single study (data not shown). Funnel plot and Egger's test were conducted in five comparison models to assess the publication bias in the overall combined meta-analyses. The shape of funnel plots did not reveal any evidence of asymmetry (Figure 3). Stratified analysis in Asian, population-based and Chinese studies also showed similar trends in the shape of the funnel plots (figures not shown). Furthermore, Egger's test in overall, Asian, population-based and Chinese studies did not show evidence of publication bias in any of the comparison models as *P*-values were larger than 0.05 (Table 4). However, publication bias (Funnel plot and Egger's test) was not possible in Caucasian and hospital-based studies because the numbers of studies were less than three. Heterogeneity within and among different studies were tested with *Q*-value, *P*-value of heterogeneity (*P*_H_) and *I*^2^ statistics (Table 4). The random-effects model was used for meta-analysis if the *Q*-statistic was significant (*P*_H_<0.05), which indicates heterogeneity across studies. The fixed-effect model was employed when P_H_⩾0.05. In the overall population, the fixed-effect model was employed for meta-analysis of the p53 codon72 Arg>Pro polymorphism in four comparison models (Pro vs Arg, ProPro vs ArgArg, ArgPro vs ArgArg and ProPro+ArgPro vs ArgArg). However, the ProPro vs ArgArg+ArgPro comparison model showed heterogeneity among studies in the overall population and the random-effect model was used.

In the stratified analysis, the fixed-effect model was employed in all comparison models of Asian studies except the Pro vs Arg comparison, in which the random-effect model was used. In Caucasian studies the fixed-effect model was employed in all comparison models. In population-based and Chinese studies the fixed-effect model was employed in all comparison models except in the ProPro vs ArgArg+ArgPro comparison model, which reflects the combined results of the overall study. In hospital-based studies the fixed-effect model was employed in three comparison models (Pro vs Arg, ProPro vs ArgArg and ProPro vs ArgArg+ProPro) and the random-effect model in two comparison models (ArgPro vs ArgArg and ProPro+ArgPro vs ArgArg). The overall pooled results indicate that the p53 codon72 polymorphism is a significant risk factor in the pathogenesis of NPC. Stratified analyses in Asian, Caucasian, hospital-based, population-based and Chinese case–control studies corroborate this association. This meta-analysis supports the findings in north-eastern Indian populations. To our knowledge, the current study is the first to analyze the p53 codon72 polymorphism and association with NPC in the Indian population.

In conclusion, our case–control study in North Indian populations and meta-analysis results as evidenced from five genetic models suggest that the p53 codon72 Arg>Pro polymorphism could be employed as a risk factor for NPC. However, some limitations exist in the current meta-analysis. Association of p53 codon72 polymorphism with susceptibility to the histological and clinical grade of NPC patients has not been investigated because of lack of available data on the subject.

The p53 Arg form is more susceptible to degradation than the Pro form by human papilloma virus E6 protein.^49^ Notably, Epstein–Barr virus infection modulates the effect of the p53 family^22^ and is a well-nown risk factor for NPC. Nevertheless, whether the p53 Arg or the Pro form is also susceptible to degradation by viruses or by other infectious agents needs to be investigated. Further, as there are a large number of SNPs for p53, the SNP studied in the present analysis was limited only to the functionally important one. In future, screening of all p53 and related polymorphisms in larger samples based on ethnicity in view of confounding factors such as age, sex, cigarette smoke, tobacco use, alcohol intake, dietary habit, stages of NPC and socioeconomic status is required to validate the findings.

## Figures and Tables

**Table 1:** Baseline and clinical characteristics of NPC cases and healthy controls

	*Cases (*n*=70)*	*Control (*n*=70)*
*General history*		
* *Age in year (⩽30/31–50/>50)	7/38/25	5/42/23
* *Gender (male/female)	46/24	49/21
* *Marital status (married/unmarried/widow)	48/18/4	42/28/0
Geographical region/ethnicity	North-east India/Asian	North-east India/Asian
		
*Specific clinical symptoms*		
* Ear*		
* *Ache	28	Nil
* *Deafness	23	Nil
* *Infection	12	Nil
* *Tinnitus	12	Nil
		
* Eye*		
* *Diplopia	14	Nil
* *Loss of vision	6	Nil
* *Protrusion of eye ball	2	Nil
* *Neck	27	Nil
		
*Swelling*		
* Nasal*		
* *Obstruction	21	Nil
* *Bleeding	20	Nil
* *Congestion	16	Nil
		
* Clinical examination*		
* *Histopathology^a^ (keratinizing squamous cell carcinoma/non-keratinizing differentiated carcinoma/undifferentiated carcinoma)	39/13/18	NE
		
* TNM staging*^b^		
* *stage 0: T_is_, N_0_, M_0_	0	NE
* *stage I: T_1_, N_0_, M_0_	4	NE
* *stage II: T_2_, N_0_, M_0_ (or T_1_ /T_2_, N_1_, M_0_)	27	NE
* *stage III: T_3_, N_0_ to N_2_, M_0_ (or T_1_ /T2, N_2_, M_0_)	16	NE
* *stage IVA: T_4_, N_0,_ N_1_/ N_2_, M_0_	21	NE
* *stage IVB: any T, N_3_, M_0_	2	NE
* *stage IVC: any T, any N, M_1_	0	NE

Abbreviations: M, metastasis; N, lymphnode; NE, not examined; T, tumor. Data are number of participants unless otherwise specified. The subjects are marked. A total of 70 NPC patients were enrolled from seven medical centers spread across the states of north-east India: (i) Dr B Borooah Cancer Institute, Guwahati, Assam; (ii) Cachar Cancer Hospital & Research Centre, Silchar, Assam; (iii) Civil Hospital, Aizawl, Mizoram; (iv) Civil Hospital, Dimapur, Nagaland; (v) Regional Institute of Medical Sciences, Imphal, Manipur; (vi) Arunachal State Hospital, Arunachal Pradesh; and (vii) Guwahati Medical College & Hospital, Guwahati, Assam. Controls and patient samples were characterized by considering their general history, geographical region, ethnicity and body symptoms. Clinical examinations of all patient samples based on World Health Organization (WHO) and AJCC classification were performed to determine the different stages of NPC. TNM, tumor, node and metastasis.

a According to the WHO histopathological grading system

.

b According to AJCC (American Joint Committee on Cancer) classification to determine different stages of NPC.

**Table 2:** Genotyping and distribution of p53 codon72 Arg>Pro polymorphism in NPC cases and healthy controls of north-eastern Indian populations

*Genotype or allele*	*Case (*n*=70)*	*Control (*n*=70)*	P*-value*	*OR (95% CI)*
*Genotype*				
ArgArg	14 (20)	34 (48.57)	1	Ref.
ArgPro	33 (47.14)	20 (28.57)	0.03	0.39 (0.18–0.97)
ProPro	23 (32.86)	16 (22.86)	0.008	0.28 (0.11–0.69)
				
*Allele*				
Arg	61 (43.57)	88 (62.86)	1	Ref.
Pro	79 (56.43)	52 (37.14)	0.001	0.45 (0.28–0.73)

Abbreviations: CI, 95% confidence interval; OR, odds ratio. Data are number (%) of participants unless otherwisespecified. For genotyping, blood samples collected from each individual were processed and genomic DNA was extracted using the GenElute Blood Genomic DNA Kit (Sigma, St Louis, MO, USA; cat no. NA2020). PCR for genotyping of p53 codon72 Arg>Pro polymorphisms was performed as described earlier.^50^ Primers were obtained from Integrated DNA Technologies (Coralville, IA, USA): one pair of primers (p53 codon72 Arg Forward: TCC CCC TTG CCG TCC CAA; P53 codon72 Arg Reverse: CTG GTG CAG GGG CCA CGC) specific for the Arg allele and the other pair (p53 codon72 Pro Forward: GCC AGA GGC TGC TCC CCC, p53 codon72 Pro Reverse: CGT GCA AGT CAC AGA CTT) for the Pro allele. PCR was performed using a PCR amplification kit (cat no. RO11; TaKaRa, Shiga, Japan) with the following reaction conditions: genomic DNA extracted from blood was amplified in a PCR reaction containing 1 × PCR buffer, 200 μm of each dNTP, 10 pmole of each primer and 0.5 unit of Taq polymerase in a final volume of 20 μl. The detection of the two polymorphic variants was carried out in two separate tubes. The amplification was performed as follows: initial denaturation at 94 °C for 3 min, amplification for 35 cycles at 94 °C for 30 s, at 60 °C for the Arg allele and at 54 °C for the Pro allele for 30 s, extension at 72 °C for 30 s, followed by a final extension at 72 °C for 5 min. The PCR product obtained was 141 bp for the Arg allele and 177 bp for the Pro allele. Heterozygous samples showed the presence of both PCR products, whereas homozygous samples exhibited only one of the two products. In each PCR reaction one blank sample containing water in place of genomic DNA was taken as the negative control. Fisher's exact test was used to examine the distribution of allele and genotype frequencies among NPC patients and healthy controls.^51^

**Table 3:** Main characteristics of studies included in the meta-analysis

***First author*** ***(year), ref.***	***Country***	***Ethnicity***	***Study*** ***design***	***Genotyping*** ***method***	***Sample size***	***Genotype (control)***			***Genotype (case)***			***MAF***	***HWE for control***		***Power (%)***
					*Control/case*	*ArgArg*	*ArgPro*	*ProPro*	*ArgArg*	*ArgPro*	*ProPro*	*Control/case*	*χ2*	P-*value*	
Present study (2014)	India	Asian	HB	AS-PCR	70/70	34	20	16	14	33	23	0.37/0.56	10.54	0.001	23
Zhang (2014)^30^	China	Asian	PB	PCR-RFLP	477/566	130	229	118	133	292	141	0.48/0.50	0.73	0.39	97
Li (2013)^28^	China	Asian	PB	PCR-RFLP	360/217	125	186	49	73	113	31	0.39/0.40	2.39	0.12	80
Xiao (2010)^29^	China	Asian	PB	PCR-RFLP	712/522	226	366	120	117	270	135	0.42/0.51	1.88	0.17	99
Hadhri-Guiga (2007)^27^	Tunisia	Caucasian	PB	PCR-RFLP	83/115	32	45	6	44	48	23	0.34/0.40	3.39	0.06	32
Sousa (2006)^26^	Portugal	Caucasian	PB	AS-PCR	285/107	178	93	14	62	32	13	0.21/0.27	0.16	0.68	61
Tiwawech (2003)^25^	Thailand	Asian	PB	PCR-RFLP	148/102	50	70	28	24	52	26	0.42/0.50	0.15	0.69	40
Tsai (2002)^24^	China	Asian	HB	PCR-RFLP	59/50	25	26	8	20	14	16	0.35/0.46	0.08	0.76	18
Yung (1997)^23^	China	Asian	PB	PCR-RFLP	31/20	10	13	8	6	11	3	0.46/0.42	0.77	0.39	10
Brigander (1997)^32^	China	Asian	PB	PCR-RFLP	105/73	31	49	25	16	31	26	0.47/0.56	0.42	0.51	29

Abbreviations: AS, allele specific; HB, hospital based; HWE, Hardy–Weinberg equilibrium; MAF, minor allele frequency; PB, population based; RFLP, restriction fragment length polymorphism. The study design based on samples collected from hospitals or random populations, different countries and ethnicities, power of the study, genotyping method and the distribution of the genotype among NPC and controls were listed. HWE was tested using the web-based tools (http://www.oege.org/software/ we-mr-calc.shtml). Power analysis was performed by G power software (version 3.1).^52^

**Table 4:** Summary of overall and stratified meta-analysis results

***Comparisons***	***Heterogeneity***			***Model***	***Forest plot analysis***			***Egger's regression analysis***		
	Q*-value*	P_*H*_	I^*2*^*(%)*		*OR*	*95% CI*	P*_OR_*	*Intercept*	*95% CI*	P*-value*
*Overall studies*										
Pro vs Arg	15.98	0.06	43.69	Fixed	1.28	1.17–1.40	<0.001	0.69	−1.53 to 2.91	0.49
ProPro vs ArgArg	16.23	0.06	44.56	Fixed	1.70	1.41–2.04	<0.001	0.75	−1.45 to 2.96	0.45
ArgPro vs ArgArg	14.95	0.09	39.80	Fixed	1.24	1.07–1.43	0.004	−0.02	−2.33 to 2.28	0.98
ProPro+ArgPro vs ArgArg	13.55	0.13	33.60	Fixed	1.35	1.17–1.55	<0.001	0.29	−1.94 to 2.54	0.76
ProPro vs ArgArg+ArgPro	19.05	0.02	52.76	Random	1.54	1.18–2.01	0.002	1.005	−1.20 to 3.21	0.32
										
*Asian studies*										
Pro vs Arg	15.78	0.02	55.65	Random	1.30	1.10–1.54	0.002	0.68	−2.24 to 3.60	0.58
ProPro vs ArgArg	13.94	0.05	49.79	Fixed	1.63	1.34–1.97	<0.001	0.37	−2.50 to 3.24	0.76
ArgPro vs ArgArg	11.40	0.12	38.62	Fixed	1.31	1.11–1.53	0.001	0.38	−2.15 to 2.93	0.72
ProPro+ArgPro vs ArgArg	12.18	0.09	42.53	Fixed	1.39	1.20–1.62	<0.001	0.58	−2.07 to 3.24	0.61
ProPro vs ArgArg+ArgPro	13.40	0.06	47.79	Fixed	1.35	1.15–1.59	<0.001	0.32	−2.42 to 3.06	0.78
										
*Caucasian studies*										
Pro vs Arg	0.02	0.87	<0.001	Fixed	1.35	1.03–1.77	0.02	**—**	**—**	**—**
ProPro vs ArgArg	0.005	0.94	<0.001	Fixed	2.71	1.44–5.09	0.002	**—**	**—**	**—**
ArgPro vs ArgArg	0.36	0.54	<0.001	Fixed	0.89	0.61–1.31	0.58	**—**	**—**	**—**
ProPro+ArgPro vs ArgArg	0.22	0.63	<0.001	Fixed	1.13	0.79–1.61	0.50	**—**	**—**	**—**
ProPro vs ArgArg+ArgPro	0.08	0.77	<0.001	Fixed	2.88	1.57–5.29	0.001	**—**	**—**	**—**
										
*Population-based studies*										
Pro vs Arg	10.42	0.16	32.82	Fixed	1.24	1.13**–**1.37	<0.001	−0.06	−2.71 to 2.58	0.95
ProPro vs ArgArg	12.99	0.07	46.11	Fixed	1.62	1.34**–**1.97	<0.001	0.27	−2.56 to 3.11	0.82
ArgPro vs ArgArg	5.42	0.60	<0.001	Fixed	1.21	1.04**–**1.41	0.01	−0.57	−2.52 to 1.38	0.49
ProPro+ArgPro vs ArgArg	5.91	0.55	<0.001	Fixed	1.31	1.13**–**1.51	<0.001	−0.43	−2.54 to 1.66	0.62
ProPro vs ArgArg+ArgPro	16.43	0.02	57.39	Random	1.46	1.09**–**1.96	0.01	0.69	−2.26 to 3.65	0.58
										
*Hospital-based studies*										
Pro vs Arg	0.90	0.34	<0.001	Fixed	1.88	1.31–2.69	0.001	—	—	—
ProPro vs ArgArg	0.23	0.63	<0.001	Fixed	3.02	1.54–5.94	0.001	—	—	—
ArgPro vs ArgArg	8.35	0.004	88.02	Random	1.65	0.28–9.48	0.57	—	—	—
ProPro+ArgPro vs ArgArg	5.06	0.02	80.25	Random	2.04	0.61–6.83	0.24	—	—	—
ProPro vs ArgArg+ArgPro	0.93	0.33	<0.001	Fixed	2.07	1.15–3.73	0.01	—	—	—
										
*Chinese studies*										
Pro vs Arg	10.16	0.07	50.80	Fixed	1.23	1.11–1.36	<0.001	−0.17	−4.00 to 3.64	0.90
ProPro vs ArgArg	10.66	0.05	53.10	Fixed	1.54	1.25–1.89	<0.001	−0.31	−4.26 to 3.64	0.83
ArgPro vs ArgArg	3.76	0.58	<0.001	Fixed	1.23	1.04–1.45	0.015	−0.80	−2.89 to 1.29	0.34
ProPro+ArgPro vs ArgArg	4.56	0.47	<0.001	Fixed	1.31	1.12–1.54	0.001	−0.55	−3.11 to 2.00	0.58
ProPro vs ArgArg+ArgPro	13.03	0.02	61.63	Random	1.36	0.97–1.90	0.07	0.14	−4.10 to 4.39	0.92

Abbreviations: 95% CI, 95% confidence intervals; Fixed, fixed-effect model; OR, odds ratio; *P*_*H*_, *P-*vaue of heterogeneity analysis. Meta-analysis was performed with comprehensive meta-analysis V2 software in overall studies, Asian, Caucasian, population-based, hospital-based and Chinese studies. Association of p53 codon72 Arg>Pro polymorphisms with NPC was assessed by the estimation of the combined odds ratio (OR), *P-*value and 95% confidence interval (CI) in five different models: (i) allele contrast (Pro vs Arg), (ii) homozygous comparison (ProPro vs ArgArg), (iii) heterozygous comparison (ArgPro vs ArgArg), (iv) dominant (ProPro+ArgPro vs ArgArg) and (v) recessive (ProPro vs ArgArg+ProPro) model. Heterogeneity between studies was calculated using Cochran's *Q*-statistic and *I*^2^ values as described earlier.^53, 54^ Based on heterogeneity or homogeneity among the included studies, the random (Der Simonian and Laird method) or fixed (Mantel–Haenszel's method) model was used to calculate combined OR and 95% CI. Publication bias was assessed from Egger's regression analysis.
